# Hybridizing Shear‐Stiffening Gel and Chemically‐Strengthened Ultrathin Glass Sheets for Flexible Impact‐Resistant Armor

**DOI:** 10.1002/advs.202403379

**Published:** 2024-06-28

**Authors:** Xuchao Wang, Zijing Zhang, Zhihua Liang, Haimin Yao

**Affiliations:** ^1^ Department of Mechanical Engineering The Hong Kong Polytechnic University Hung Hom Kowloon Hong Kong SAR 999077 China

**Keywords:** chemical strengthening, energy absorption, protective armor, shear stiffening

## Abstract

Traditional anti‐impact armors and shields are normally made of stiff and hard materials and therefore deficient in flexibility. This greatly limits their applications in protecting objects with complex geometries or significant deformability. Flexible armors can be developed with the application of hard platelets and soft materials, but the lower rigidity of the flexible armors renders them incapable of providing sufficient resistance against impact attacks. To address the inherent conflict between flexibility and impact resistance in traditional armors, here, a composite is developed by hybridizing a shear‐stiffening gel as the matrix and chemically‐strengthened ultrathin glass sheets (CSGS) as the reinforcement. The resulting laminate, termed PCCL, exhibits both high flexibility and high impact resistance. Specifically, at low strain rates, the high ductility of the gel combined with the high flexural strength of the CSGS enables the PCCL to undergo considerable deformation; at high strain rates, on the other hand, the shear stiffening behavior of the gel matrix endows the PCCL with excellent impact resistance manifested by its high performance in energy absorption and high rigidity. With the combination of high flexibility and high impact resistance, the PCCL is demonstrated to be an ideal armor for protecting curved vulnerable objects from impact attacks.

## Graphical abstract

High flexibility and high impact resistance are two pivotal but contradictory requirements for an ideal protective armor. Here, the conflict between them is reconciled by hybridizing a non‐Newtonian gel as the matrix and chemically strengthened ultrathin glass sheets as the reinforcement. The resulting composite laminate (PCCL) demonstrates exceptional flexibility under static load and superior resistance to dynamic impact attack.

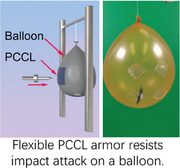

## 1 Introduction

Materials with high impact resistance are always desired when producing protective armors and shields. In ancient times, iron was the prevailing material used to develop armors for warriors.^[^ ^1^ ^]^ In recent years, advanced ceramics, which feature lighter weight, higher durability, and excellent strength and toughness,^[^ ^2^, ^3^ ^]^ were utilized to fabricate anti‐impact armors.^[^ ^4^, ^5^, ^6^ ^]^ The superior impact resistance of the metallic and ceramic armors and shields is largely attributed to the high stiffness and rigidity of the building materials, which inevitably affect their flexibility and therefore limit the application to objects with complex shapes or considerable deformability.

One conceivable strategy for flexible armors is to apply soft building materials such as high‐performance fibers, which exhibit exceptional flexibility and superior energy absorption capability.^[^ ^7^, ^8^, ^9^, ^10^, ^11^ ^]^ However, such high energy absorption mainly resulted from the large irreversible deformation of the armor. Under this circumstance, the protected objects beneath the armor might undergo considerable impact force, resulting in serious damage.^[^ ^12^, ^13^ ^]^ Another prevalent strategy for achieving flexibility is to combine rigid platelets and soft substrates to form soft‐rigid unified armors.^[^ ^14^, ^15^, ^16^, ^17^ ^]^ Although the rigid platelets can offer some impact resistance, the lower rigidity of the soft‐rigid unified armors under dynamic impact renders them incapable of providing sufficient resistance force against the impactor. This is a common issue encountered by most current flexible armors, which limits their application in protecting vulnerable objects. Developing flexible armors with high impact resistance remains a challenge calling for new strategies.

**Figure 1:**
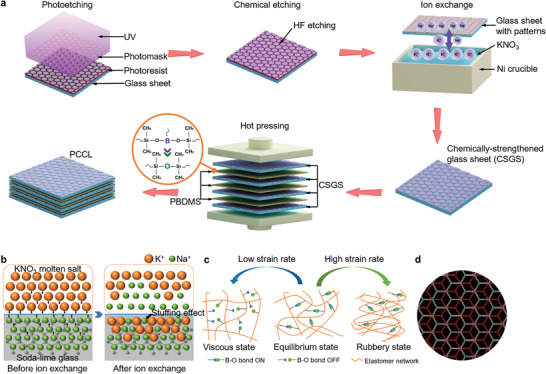
Design strategy of PCCL. a) Schematic illustration of the fabrication process of PCCL. b) Schematic illustration of the ion exchange process for strengthening ultrathin glass sheets. After the ion exchange, a compressive residual stress field is formed near the surface of the glass sheet, due to the replacement of the sodium ions (Na^+^) by the larger potassium ions (K^+^). c) Schematic illustration showing the mechanism accounting for the shear stiffening effect of PBDMS. At low strain rates, the B‐O dynamic bonds can be dissociated easily, resulting in a viscous gel‐like behavior. In contrast, at high strain rates, the B‐O dynamic bonds are associated firmly, leading to the crosslink of elastomer chains and high rigidity of the PBDMS. d) Schematic illustration showing the staggered stacking pattern of the CSGS.

Essentially, flexibility and impact resistance are mechanical properties exhibited by materials at low and high strain rates, respectively. To achieve high flexibility at a low strain rate and high rigidity (high impact resistance) at a high strain rate, materials with rate‐dependent mechanical behavior should be given high priority. This draws our attention to shear stiffening materials, also known as non‐Newtonian viscous materials, which exhibit high flexibility at low strain rate while high rigidity at high shear strain rate.^[^ ^18^, ^19^, ^20^, ^21^, ^22^ ^]^ However, most non‐Newtonian materials flow even under an unstressed state, which limits their application as structural materials unless reinforcing phase is applied.^[^ ^23^, ^24^, ^25^, ^26^ ^]^ To maintain high flexibility after the reinforcement, an ideal reinforcing phase should be able to sustain considerable deformation as well. This reminds us of the chemically‐strengthened ultrathin glass sheets (CSGS). Although bulk glass is deemed brittle and fragile, ultrathin glass sheets (≤100 µm), especially those strengthened by ion‐exchange technique, exhibit flexural strength as high as 1000 MPa.^[^ ^27^, ^28^ ^]^ That's why CSGS is currently applied to the screens of foldable cell phones. In this context, herein we propose to develop a flexible anti‐impact composite armor by using polyborodimethylsiloxane (PBDMS) as a non‐Newtonian matrix and chemically‐strengthened ultrathin glass sheets as the reinforcing phase (**Figure** **1**a). The fabrication process starts from the strengthening process of glass sheets through ion exchange (Figure 1b), followed by the deposition of PBDMS, a shear stiffening gel (Figure 1c). Finally, the chemically‐strengthened glass sheets coated with PBDMS are assembled through a staggered stacking pattern mimicking the arrangement of aragonite platelets in nacre (Figure 1d), resulting in a PBDMS/CSGS composite laminate, termed PCCL. Under a static bending load, the PCCL demonstrates exceptional flexibility. When subjected to high‐speed impact load, it displays an excellent impact resistance as reflected by high energy absorption and high rigidity, and unmatched protection potential in comparison with various control samples. With the combination of high flexibility and high impact resistance, the PCCL is demonstrated an ideal armor for protecting vulnerable objects with curved shapes, such as balloons, from damage by a fatal projectile attack.

## 2 Results and Discussion

### 2.1 Fabrication and Characterization

**Figure 2:**
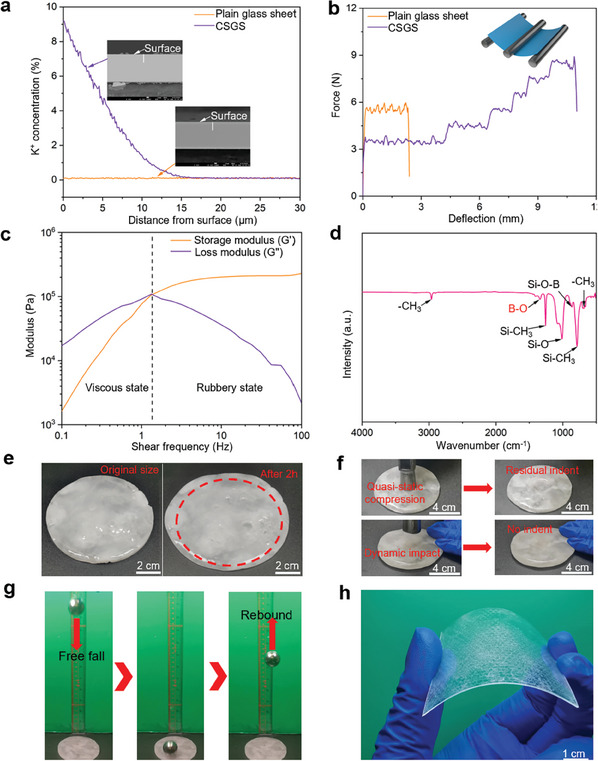
Characterization of the chemically‐strengthened glass sheets, PBDMS, and PCCL. a) K^+^ concentration profile determined by EPMA along thickness direction of a glass sheet before and after ion exchange. b) Force‐deflection curves from the 3‐point bending tests on 100 µm thick glass sheets with and without ion exchange. c) Storage modulus and loss modulus of PBDMS measured by a rheological test. d) FTIR spectrum of PBDMS showing the presence of the B─O dynamic bonds. e) Digital photograph showing the flowability of PBDMS upon gravity. f) Distinct responses of a PBDMS disc to quasi‐static compression and dynamic impact. g) Digital photograph showing the excellent resilience of PBDMS. h) Digital photograph showing the high flexibility of the PCCL.

The fabrication process of the PCCL is schematically depicted in Figure 1a. First, photoetching technology with a honeycomb‐like photomask was applied to deposit a patterned photoresist film onto 100 µm thick soda‐lime glass sheets (Figure S1a,b, Supporting Information). The width of each hexagonal photoresist film was about 2 mm (Figure S1a, Supporting Information). The glass sheets covered with patterned photoresist film were subsequently immersed in a hydrofluoric acid (HF) solution for chemical etching. Due to the protection by the photoresist, the etching takes place only in the uncovered region between the hexagonal films. After etching and removal of the photoresist, patterned hexagonal glass islands were formed on the ultrathin glass sheets (Figure S2, Supporting Information). Then, the etched glass sheets were then soaked in a molten salt of potassium nitrate (KNO_3_) at a temperature of 450 °C for 180 min. During this period, the larger‐radius potassium ions from the molten salt replaced the smaller‐radius sodium ions near the surfaces of the glass sheets.^[^ ^29^, ^30^, ^31^ ^]^ This is confirmed by both the electron probe micro‐analyzer (EPMA) and energy disperse spectroscopy (EDS) (**Figure** **2**a; Figure S3, Supporting Information). As a result, compressive residual stress is generated near the surfaces of the glass sheets.^[^ ^32^, ^33^, ^34^ ^]^ The EPMA results demonstrated that the penetration depth of the potassium ions was ≈15 µm, as depicted in Figure 2a. The compressive residual stress near the surfaces of the glass sheets can compensate for the tensile stress caused by bending load, effectively enhancing the flexural strength and flexibility of the glass sheets (Figure 2b; Figure S4 and Movie S1, Supporting Information).

The PBDMS gel was developed by reacting boric acid (H_3_BO_3_) and hydroxyl‐terminated polydimethylsiloxane (PDMS‐OH).^[^ ^7^, ^35^ ^]^ The shear stiffening behavior of the resulting PBDMS gel was confirmed by the rheology experiment (Figure 2c). In the low‐frequency region (below 1.4 Hz), the loss modulus (G″) of PBDMS is greater than the storage modulus (G′), suggesting that viscosity is predominant.^[^ ^23^, ^36^ ^]^ In contrast, as the shear frequency increases, the storage modulus surpasses the loss modulus beyond the cross point, indicating that the material behavior is dominated by the elastic solid state. Such shear stiffening behavior of PBDMS can be attributed to the presence of B─O dynamic bonds,^[^ ^9^, ^25^, ^35^ ^]^ as identified by the Fourier transform infrared spectroscopy (FTIR) result (Figure 2d). In polydimethylsiloxane (PDMS), however, such B─O dynamic bonds are deficient and therefore no shear stiffening behavior can be observed (Figure S5, Supporting Information). The unique shear stiffening behavior of PBDMS endows it with high ductility and flexibility at low strain rates, while high rigidity and impact resistance at high strain rates (Figure 2e–g; Movie S2, Supporting Information). The chemically‐strengthened glass sheets were immersed in a 5 mg mL^−1^ PBDMS‐acetone solution for 8 h. The mass fraction of PBDMS on glass sheets was controlled to be ≈5 wt%, akin to the mass fraction of organic biopolymers in nacre.^[^ ^37^, ^38^ ^]^ The glass sheets with PBDMS were stacked in a staggered manner, as shown in Figure 1d. Finally, a hot‐pressing process was employed under a vacuum atmosphere to form tight bonding between the glass sheets and the PBDMS interlayers, resulting in a PCCL with dimensions of 60 × 60 × 0.55 mm^3^ (Figure S6, Supporting Information). The dimensions of PCCL can be customized easily by altering the size of the ultrathin glass sheets or the number of layers stacked. The resultant PCCL was demonstrated capable of sustaining large flexural deformations (Figure 2h).

### 2.2 Flexibility at Low Strain Rates

To characterize the flexibility of PCCL, we carried out quasi‐static flexural tests on a PCCL specimen. To shed light on the effects of the shear stiffening gel (PBDMS) and chemical strengthening glass, we adopted two control samples. One is a soda‐lime glass panel, and another is a composite laminate called Quasi‐PCCL, which has the same compositions and structure as the PCCL except that its ultrathin glass sheets have not been strengthened. For a better comparison, all three samples are made into the same dimensions.

**Figure 3:**
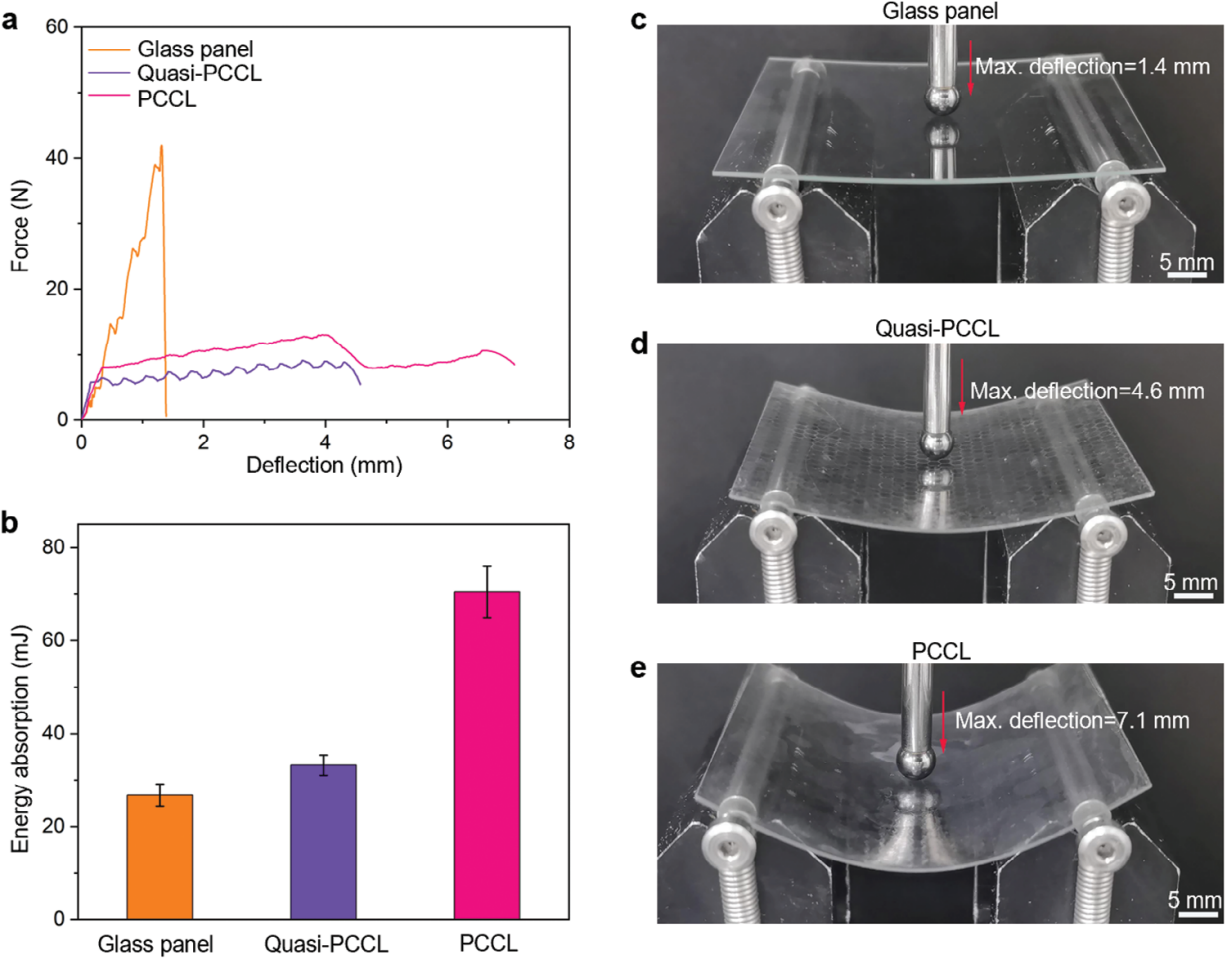
Flexibility of PCCL at low strain rates. a) Force‐deflection curves obtained from quasi‐static bending tests on three samples. b) Comparison of the energy absorption in the bending tests for a glass panel, a Quasi‐PCCL, and a PCCL. Here, the energy absorption is calculated from the area beneath the force‐deflection curve of the bending test. c–e) Snapshots at the maximum deflection moments in the bending tests performed on (c) a glass panel, d) a Quasi‐PCCL, and e) a PCCL.

The glass panel exhibits limited deformability and a typical brittle fracture mode under bending (**Figure** **3**a,c). At the peak load, cracks originate from the loading point and propagate quickly, resulting in catastrophic damage of the specimen (Movie S3, Supporting Information). In contrast, both composite laminates, including PCCL and Quasi‐PCCL, manifest higher flexibility as evidenced by the larger deflection before catastrophic damage (Figure 3a,d,e; Movie S3, Supporting Information). This can be attributed to the large deformability of the PBDMS matrix and the ultrathin flexible glass sheets as well as the laminated structure. Comparison between these two composite laminates shows that the PCCL is superior in both flexibility and energy absorption (Figure 3b). Such superiority of PCCL is attributed to the enhanced flexural strength of the ultrathin glass sheets after ion exchange.^[^ ^27^ ^]^ Comparing the morphologies of the damaged specimens after the bending tests shows that the glass sheets in the PCCL shatter into many small pieces (Figure S7, Supporting Information). This results from the severe residual stress developed in the chemically‐strengthened glass which will facilitate the fracture of the glass once the compressive surface layer is damaged,^[^ ^27^, ^39^ ^]^ akin to the explosive disintegration of a “Prince Rupert's drops” when the tail is broken.

### 2.3 Resistance to Dynamic Impact

**Figure 4:**
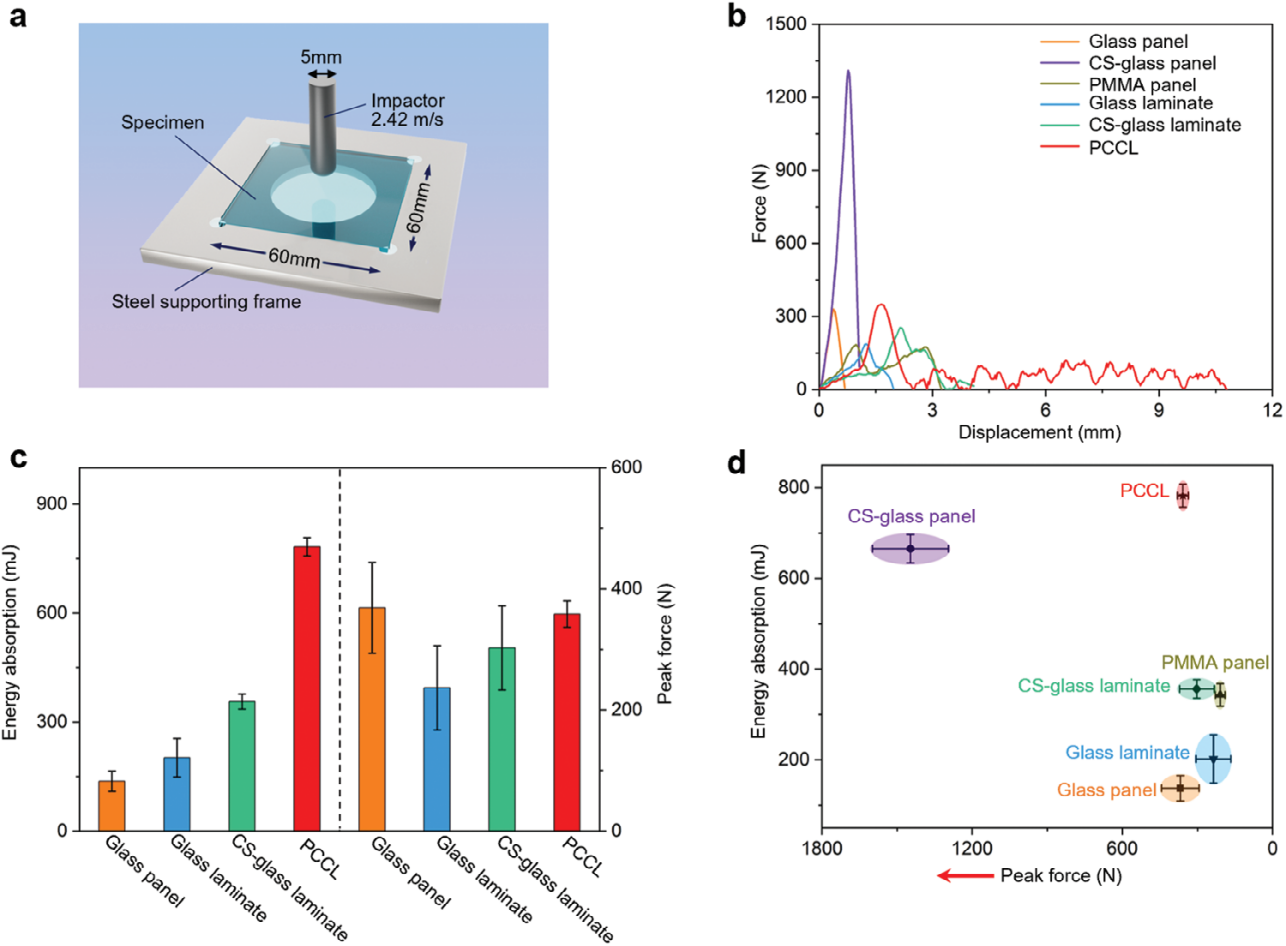
Investigation of the impact resistance of PCCL at high strain rates. a) Schematic illustration of drop‐hammer impact test. b) Force‐displacement curves under drop‐hammer impact tests. c) Comparison of energy absorption and peak force for glass panel, glass laminate, CS‐glass laminate, and PCCL under drop‐hammer impact tests. d) Ashby plot (energy absorption versus peak reaction force) for PCCL and different control samples under the drop‐hammer impact tests.

To assess the impact resistance of the PCCL, we conducted drop‐hammer impact tests on a PCCL specimen (60 mm × 60 mm × 2 mm) and a series of control samples of the same dimensions by adopting the same impactor and drop height (**Figure** **4**a). The control samples include a soda‐lime glass panel (Glass panel), a chemically strengthened glass panel (CS‐glass panel), a polymethyl methacrylate panel (PMMA panel), a composite laminate composed of 100 µm thick glass sheets and PBDMS matrix (Glass laminate), a composite laminate composed of chemically‐strengthened 100 µm thick glass sheets and PBDMS matrix (CS‐glass laminate). The fraction of PBDMS in the glass laminate, CS‐glass laminate and PCCL is controlled the same (5%). The results indicate that both the glass panel and CS‐glass panel display typical brittle fracture behavior (Figure 4b). In contrast, the PMMA panel, the glass laminate, the CS‐glass laminate and the PCCL display features of ductile fracture under dynamic impact load. Particularly, the ductility of the PCCL significantly surpasses those of the control specimens, indicating its high energy absorption capability.^[^ ^8^, ^40^ ^]^ Among all the samples, the glass panel exhibits the lowest energy absorption (Figure 4c). The glass laminate outperforms the glass panel in energy absorption, owing to the enhanced flexibility brought about by the laminated design and the increased storage modulus of PBDMS at high strain rates. Furthermore, the CS‐glass laminate surpasses the glass laminate in energy absorption (Figure 4c), implying that ion exchange is an effective approach to enhance the impact resistance. This can be attributed to the higher flexural strength brought about by the ion exchange process.^[^ ^41^, ^42^ ^]^ The PCCL demonstrates an energy absorption more than two folds of that of the CS‐glass laminate (Figure 4c). Such a significant enhancement in impact resistance should be ascribed to the hexagonally grooved structure which can branch the crack propagation and therefore result in higher energy consumption.^[^ ^15^, ^43^, ^44^ ^]^ In addition, the PCCL also exhibits superior energy absorption as compared with the CS‐glass panel and PMMA panel (Figure S8, Supporting Information). Among all the tested samples, the CS‐glass panel displays the highest peak force as depicted by Figure 4b, while the PMMA panel shows the lowest peak force (Figure S8, Supporting Information). It is interesting to notice that the peak force of the PCCL is comparable to that of the glass panel. This implies the high rigidity of the PCCL under dynamic load, which endows it with an excellency in protecting vulnerable objects against impact attack. To manifest the effect of shear stiffening, we replaced the PBDMS in the CS‐glass laminate with the similar amount of PDMS, which is deficient of shear stiffening mechanism. The resulting composite exhibits much reduced peak force and energy absorption as compared to the CS‐glass laminate (Figure S9, Supporting Information), implying the positive contribution of the shear stiffening mechanism to the impact resistance. The shear stiffening mechanism of PBDMS originates from the dynamic crosslinks of B─O in it. At low strain rates, the B─O crosslinks have enough time to respond to external stimuli, and can be easily associated and dissociated, which endows the PBDMS with a viscous gel‐like behavior at the macroscale.^[^ ^45^, ^46^, ^47^ ^]^ In contrast, at high strain rates, the B─O crosslinks cannot respond to the external stimuli in time and this may hinder the movement of molecular chains. Under this circumstance, PBDMS behaves like an elastic solid with considerable rigidity. The high energy absorption and high rigidity under impact load in combination with its high flexibility under static load make the PCCL an ideal candidate for protective armor (Figure 4d).

**Figure 5:**
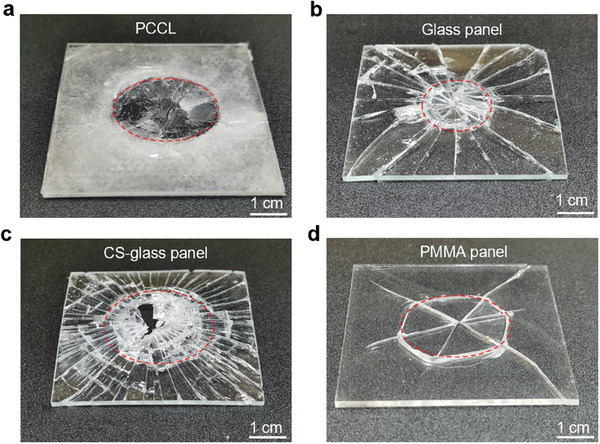
Digital photographs after drop‐hammer impact tests on a) PCCL, b) glass panel, c) CS‐glass panel, and d) PMMA panel. The red dashed lines indicate the smashed region of the sample.

To gain more insights into the high energy‐absorption capability of the PCCL, we further compared the fracture patterns of some samples after the impact tests (**Figure** **5**). For the PCCL, which exhibited the highest energy absorption among the samples, the impact left an apparent circular region where the glass was severely smashed (Figure 5a). The diameter of the smashed region is ≈3 cm, which is more than two times of the impactor's diameter. Out the smashed regions, the structural integrity of the sample was well maintained. The considerable energy consumption in forming such a large region accounts for the high energy absorption of the PCCL. In contrast, for the glass panel manifesting the lowest energy absorption in the tests, the impact produced tens of radical cracks as well as a smashed region ≈2 cm in diameter (Figure 5b). Such a smaller smashed region in combination with the lower crack density and the lower fracture toughness of glass determines its lower energy absorption. For the CS‐glass panel, however, the impact generated a similar fracture pattern but a considerably smashed region with a much higher crack density (Figure 5c). This explains why the CS‐glass panel exhibited an energy absorption comparable to that of the PCCL (Figure 4d). For the PMMA panel, the impact left only six radial cracks and one ring crack. Four radial cracks terminated inside the sample (Figure 5d). Although PMMA has a higher fracture toughness than glass, such a short crack length fails to bring high energy absorption to the PMMA panel under impact (see Figure 4d). It is interesting to notice that the impact tests caused catastrophic radial cracks on all samples except the PCCL. This might be attributed to the microscopic hexagonal grooves on the glass sheet, which lead to crack branching into diverse directions before stopping propagating.

**Figure 6:**
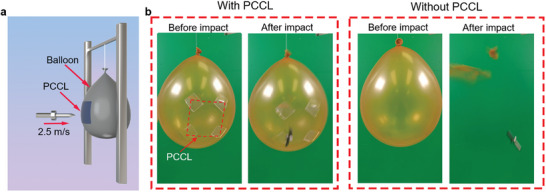
The demonstration of the potential application of the PCCL in protecting a vulnerable balloon. a) Schematic illustration showing the set‐up of the projectile test. b) Projectile attack tests on a latex balloon with or without protection by a PCCL.

The combination of high flexibility and high impact resistance exhibited by the PCCL endows it with the unique potential in some applications such as protective armor for objects with curved shapes. To demonstrate this point, we attached a PCCL (60 mm × 60 mm × 0.55 mm) to the surface of a latex balloon and carried out projectile attacks with a dart (**Figure** **6**a). With the protection of the PCCL panel which perfectly conforms to the balloon's surface, the vulnerable balloon can survive from the fatal projectile attacks by the dart (Figure 6b; Movie S4, Supporting Information).

## 3 Conclusion

To summarize, in this paper we applied a shear‐stiffening gel and chemically‐strengthened glass sheets to develop a composite laminate, called PCCL, to address the inherent conflict between flexibility and impact resistance in the traditional anti‐impact armors. At low strain rates, the resulting PCCL demonstrated considerable flexibility, which can be attributed to two factors: i) the enhanced flexural strength of the ultrathin glass sheets after ion exchange process; and ii) the flowable gel state of PBDMS at low strain rates. At high strain rates, the PCCL demonstrated excellent impact resistance evidenced by its high energy absorption and high rigidity under impact attacks. Such a high impact resistance can be ascribed to the shear stiffening behavior of the PBDMS matrix and the high flexural strength of the chemically strengthened glass sheets. With the possession of both high flexibility and high impact resistance, our PCCL has been demonstrated to have great application potential as an anti‐impact armor for objects with curved profiles.

## 4 Experimental Section

### Preparation of Hexagonal Patterns in Ultrathin Glass Sheets

The ultrathin glass sheets were supplied by Asahi Glass Co., Ltd. (Japan). The compositions were confirmed by X‐ray fluorescence (XRF, SHIMADZU XRF‐1800, Japan), and the result is shown in Table S1 (Supporting Information). The ultrathin glass sheets were first cleaned in acetone and then dried in a drying oven. Subsequently, AZ 5214E photoresist (MicroChemicals GmbH, Ulm, Germany) was spin‐coated (Sawatec Spin Coater, Switzerland) onto the cleaned ultrathin glass sheets. The spin speed was 4000 rpm and the spin time was 40s, in this case, a 3.2 µm thick photoresist layer was coated onto the ultrathin glass sheet (Figure S1b, Supporting Information). Then, the ultrathin glass sheets were transferred to a hot plate to improve adhesion between the photoresist and glass sheets under 120 °C for 2 min. To get photoresist patterns, the ultrathin glass sheets were transferred to a UV mask aligner (SUSS MA6 Mask Aligner, Germany), under the effect of UV light (Wavelength: 365 nm, Power: 350 W, Exposure time: 20 s), patterns from the photomask were transferred to the photoresist on the ultrathin glass sheets. After developing in AZ 300 MIF developer, the hexagonal photoresist patterns presented on the ultrathin glass sheets (Figure S1, Supporting Information). To further transfer the photoresist patterns into ultrathin glass sheets, a chemical etching was implemented. To be specific, the ultrathin glass sheets were immersed in HF solution for 20 min. It is noteworthy that the HF solution was obtained by mixing ammonium bifluoride, 98% sulfuric acid, 37% hydrochloric acid, and deionized water. After cleaning and drying, ultrathin glass sheets with hexagonal patterns were obtained (Figure S2, Supporting Information).

### Ion Exchange

The ion exchange processing was performed in a muffle furnace (KSL‐1200X, Hefei Kejing Materials Technology Co., Ltd., China). The potassium nitrate (KNO_3_, Purity ≥ 99%, Sigma–Aldrich Co. LLC, USA) in a nickel crucible was first heated to 450 °C at a heating rate of 10 °C min^−1^ to get KNO_3_ molten salt. During this procedure, the ultrathin glass sheets with hexagonal patterns placed in a molybdenum mesh basket were held above a nickel crucible and heated together with KNO_3_. Then, the glass sheets were immersed into KNO_3_ molten salt to conduct ion exchange at 450 °C for 180 min. After that, the glass sheets were withdrawn from the molten salt and cooled to room temperature with the muffle furnace. Subsequently, the ion‐exchanged glass sheets were cleaned using distilled water to remove residual salt on the surfaces of the glass sheets.

### Preparation of PBDMS

First, boric acid (H_3_BO_3_, Shanghai Aladdin Biochemical Technology Co., Ltd., China) and hydroxyl‐terminated polydimethylsiloxane (PDMS‐OH, Shanghai Bide Pharmaceutical Technology Co., Ltd., China) were mixed and stirred at a mass ratio of 5:95. Then, the mixture was heated to 180 °C using the muffle furnace and maintained at this temperature for 180 min. Eventually, PBDMS was obtained after cooling the resulting polymer to room temperature.

### Fabrication of PCCL

Chemically‐strengthened glass sheets and PBDMS were hybridized to prepare the PCCL. First, 5 g PBDMS was fully dissolved in 100 mL acetone to get a stable PBDMS solution. Subsequently, the chemically‐strengthened glass sheets were immersed in PBDMS solution for 8 h to obtain sufficient PBDMS adhesion. After that, the glass sheets was assembled in a staggered arrangement mode (Figure 1d). The study then used a pre‐pressing step with a pressure of 1 MPa to make different glass laminations fully contact. Finally, a vacuum hot‐pressing step was applied to improve the adhesion between the chemically‐strengthened glass sheets and PBDMS under a 5 MPa pressure for 6 h. Through the above‐mentioned procedures, the PCCL (Figure 2h) was obtained.

### Quasi‐Static Compression Tests

The specimens used for the quasi‐static compression tests were 60 mm × 60 mm × 0.55 mm panels. For each group, at least four specimens were tested. The tests were performed on a universal testing machine (MTS QTest/25, USA). A loading nose with a spherical tip (diameter: 6 mm) was driven into the center of the specimens at a quasi‐static rate of 0.5 mm min^−1^ until failure. The force and deflection curves were recorded during the tests, and the total energy absorption for each specimen was calculated according to the area of the force‐deflection curve. An optical camera was used to record the compression processes during the tests and failure morphologies after the tests.

### Drop‐Hammer Impact Tests

The dynamic drop‐hammer impact tests were performed on a drop‐weight impact testing system (Instron 9250HV, USA) equipped with a 17.45 kg drop weight and a hemispherical‐tip impactor (12.7 mm in diameter). The specimens used for the drop‐hammer impact tests were 60 mm × 60 mm × 2 mm panels. For each group, at least four specimens were tested. Before the impact tests, the specimens were clamped in a steel supporting frame (Figure 4a). During the tests, the same impact velocity of 2.42 m s^−1^ was adopted. Force‐displacement curves were plotted according to the results of the impact tests, and energy absorption for each specimen was obtained by calculating the area beneath the force‐displacement curve. After the impact tests, an optical camera was used to record the failure morphologies of the tested specimens.

### Demonstration of PCCL as a Flexible Armor

To demonstrate the efficacy of the PCCL in protecting objects with curved surfaces, a model test illustrated in Figure 6a was designed. The PCCL was attached to the surface of a balloon, a tungsten carbide dart with a tip was used to impact the balloon protected by the PCCL. For comparison, the dart was also used to impact the balloon without any protection. A catapult was applied to launch the darts from the same distance to the balloons, resulting in a constant impacting speed of around 2.5 m s^−1^. All balloons were inflated manually with a pump to the same size.

### Characterizations

The hexagonal photoresist patterns on ultrathin glass sheets obtained by the photoetching process were characterized using a digital microscopy system (KEYENCE VHX‐7000, Japan). The patterns in ultrathin glass sheets obtained by the chemical etching process were characterized using an optical microscope (Nikon EPIPHOT 200, Japan) and an optical profiler (New Zygo NexView, USA). The concentration of potassium ion was characterized using an electron probe micro‐analyzer (EPMA, JXA‐8530F PLUS, Japan) and an energy disperse spectroscopy (EDS, X‐max, UK). The flexural strength and flexibility of the plain glass sheet and the chemically‐strengthened glass sheet were characterized by a three‐bending method, and the span and loading rate were 50 mm and 0.5 mm min^−1^, respectively. The rheological properties were characterized using a rheometer (Haake Mars60, Germany). Fourier transform infrared spectroscopy (FTIR) data of PBDMS was obtained by a Thermo Scientific iN10 spectrometer (USA) in the wavenumber range of 4000–500 cm^−1^.

## Conflict of Interest

The authors declare no conflict of interest.

## Author Contributions

H.Y. and X.W. conceived the idea and designed the experiments. H.Y. supervised the research. X.W., Z.Z., and Z.L. performed the fabrication and characterization. X.W. analyzed the data. X.W. and H.Y. co‐wrote the manuscript. All authors discussed the results. All authors participated in discussions of the research.

## Supporting information

Supporting Information

Supplemental Movie 1

Supplemental Movie 2

Supplemental Movie 3

Supplemental Movie 4

## Data Availability

The data that support the findings of this study are available from the corresponding author upon reasonable request.

## References

[1] N. V. David , X. L. Gao , J. Q. Zheng , Appl. Mech. Rev. 2009, 62, 050802.

[2] A. Ghazlan , T. Ngo , P. Tan , Y. M. Xie , P. Tran , M. Donough , Compos. Part B 2021, 205, 108513.

[3] H. C. Zhang , L. P. Shi , X. L. Ma , L. Yang , Y. S. Zhong , X. D. He , J. Appl. Phys. 2022, 131, 135105.

[4] L. J. Li , L. F. Cheng , S. W. Fan , X. J. Gao , Y. P. Xie , L. T. Zhang , Mater. Design 2015, 79, 26.

[5] P. Jannotti , G. Subhash , A. K. Varshneya , J. Am. Ceram. Soc. 2014, 97, 189.

[6] C. Y. Huang , Y. L. Chen , Mater. Design 2016, 91, 294.

[7] Y. P. Chen , B. K. Dang , J. Z. Fu , J. Y. Zhang , H. Y. Liang , Q. F. Sun , T. Y. Zhai , H. Q. Li , ACS Nano 2022, 16, 7525. 35499235 10.1021/acsnano.1c10725

[8] Z. B. Zhang , Z. Z. He , X. F. Pan , H. L. Gao , S. M. Chen , Y. B. Zhu , S. S. Cao , C. Y. Zhao , S. Wu , X. L. Gong , H. A. Wu , S. H. Yu , Small 2023, 19, 2205219. 10.1002/smll.202205219 36404124

[9] J. Y. Zhou , J. S. Zhang , M. Sang , S. Liu , F. Yuan , S. Wang , S. S. Sun , X. L. Gong , Chem. Eng. J. 2022, 48, 131878.

[10] B. J. Zhang , J. D. Yang , Y. J. Li , J. Q. Zhang , S. C. Niu , Z. W. Han , L. Q. Ren , Int. J. Mech. Sci. 2023, 244, 108073.

[11] F. Qi , J. Gao , B. L. Wu , H. Y. Yang , F. G. Qi , N. Zhao , B. Zhang , X. P. Ouyang , Polymers 2022, 14, 4177.36236125

[12] F. Tang , C. Dong , Z. Yang , Y. Kang , X. C. Huang , M. H. Li , Y. C. Chen , W. J. Gao , C. G. Huang , Y. C. Guo , Y. P. Wei , Compos. Sci. Technol. 2022, 218, 109190.

[13] Q. Y. He , S. S. Cao , Y. P. Wang , S. H. Xuan , P. F. Wang , X. L. Gong , Compos. Part A 2018, 106, 82.

[14] L. Z. Mao , M. J. Zhou , L. Yao , H. Yu , X. F. Yan , Y. Shen , W. S. Chen , P. B. Ma , Y. Ma , S. L. Zhang , S. C. Tan , Adv. Funct. Mater. 2023, 33, 2213419.

[15] Z. Yin , F. Hannard , F. Barthelat , Science 2019, 364, 1260. 31249053 10.1126/science.aaw8988

[16] S. Estrada , A. Ossa , Adv. Eng. Mater. 2020, 22, 2000006.

[17] R. Martini , F. Barthelet , Bioinspir. Biomim. 2016, 11, 066001. 27736808 10.1088/1748-3190/11/6/066001

[18] X. K. Liu , C. Qian , K. J. Yu , Y. Jiang , Q. Q. Fu , K. Qian , Smart Mater. Struct. 2020, 29, 045018.

[19] W. H. Wang , S. Wang , J. Y. Zhou , H. X. Deng , S. S. Sun , T. Xue , Y. Q. Ma , X. L. Gong , Adv. Funct. Mater. 2023, 33, 2212093.

[20] C. Y. Zhao , X. L. Gong , S. Wang , W. Q. Jiang , S. H. Xuan , Cell Rep. Phys. Sci. 2020, 1, 100266.

[21] Z. Y. Fan , L. Lu , M. Sang , J. P. Wu , X. Y. Wang , F. Xu , X. L. Gong , T. Z. Luo , K. C. F. Leung , S. H. Xuan , Adv. Sci. 2023, 10, 2302412. 10.1002/advs.202302412 PMC10502653 37424041

[22] X. Zhang , J. Y. Zhou , K. J. Wu , S. S. Zhang , L. L. Xie , X. L. Gong , L. H. He , Y. Ni , Adv. Mater. 2024, 36, 2311817. 10.1002/adma.202311817 38226720

[23] L. W. Wu , F. Zhao , Z. Q. Lu , J. H. Lin , Q. Jiang , Compos. Struct. 2022, 298, 116009.

[24] X. K. Liu , K. J. Yu , Q. Q. Fu , K. Qian , Smart Mater. Struct. 2019, 28, 055017.

[25] M. Sang , J. S. Zhang , S. Liu , J. Y. Zhou , Y. Wang , H. X. Deng , J. Li , J. Li , S. H. Xuan , X. L. Gong , Chem. Eng. J. 2022, 440, 135869.

[26] F. Zhao , H. Chen , H. Gu , Q. Jiang , Z. Q. Lu , L. W. Wu , J. Ind. Text. 2022, 51, 2799S.

[27] L. Wondraczek , E. Bouchbinder , A. Ehrlicher , J. C. Mauro , R. Sajzew , M. M. Smedskjaer , Adv. Mater. 2022, 34, 2109029. 10.1002/adma.202109029 34870862

[28] S. Hödemann , A. Valdmann , J. Anton , T. Murata , J. Mater. Sci. 2016, 51, 5962.

[29] D. J. Green , R. Tandon , V. M. Sglavo , Science 1999, 283, 1295. 10037593 10.1126/science.283.5406.1295

[30] X. C. Li , M. Meng , D. Li , R. Wei , L. He , S. F. Zhang , J. Eur. Ceram. Soc. 2020, 40, 4635.

[31] V. M. Sglavo , A. Quaranta , V. Allodi , G. Mariotto , J. Non‐Cryst. Solids 2014, 401, 105.

[32] L. B. Jiang , Y. Wang , I. Mohagheghian , X. Y. Li , X. T. Guo , L. Li , J. P. Dear , Y. Yan , Mater. Design 2017, 122, 128.

[33] O. T. Sanya , S. S. Owoeye , O. J. Ajayi , J. Non‐Cryst. Solids 2018, 494, 9.

[34] M. N. Svenson , L. M. Thirion , R. E. Yongman , J. C. Mauro , S. J. Rzoska , M. Bockowski , M. M. Smedskjaer , ACS Appl. Mater. Interfaces 2014, 6, 10436. 24911917 10.1021/am5019868

[35] Z. Y. Fan , C. Y. Zhao , J. P. Wu , Y. B. Gai , J. Y. Zhou , J. S. Zhang , X. L. Gong , S. H. Xuan , Compos. Part A 2022, 161, 107078.

[36] K. Myronidis , M. Thielke , M. Kopeć , M. Meo , F. Pinto , Compos. Sci. Technol. 2022, 222, 109395.

[37] H. B. Yao , J. Ge , L. B. Mao , Y. X. Yan , S. H. Yu , Adv. Mater. 2014, 26, 163. 24338814 10.1002/adma.201303470

[38] Y. Y. Wang , S. E. Naleway , B. Wang , Bioact. Mater. 2020, 5, 745. 32637739 10.1016/j.bioactmat.2020.06.003 PMC7317171

[39] P. Jannotti , G. Subhash , A. K. Varshneya , Int. J. Impact Eng. 2015, 75, 53.

[40] S. M. Wen , S. M. Chen , W. T. Gao , Z. J. Zheng , J. Z. Bao , C. Cui , S. Liu , H. L. Gao , S. H. , Adv. Mater. 2023, 35, 202211175. 10.1002/adma.202211175 36891767

[41] P. Jannotti , G. Subhash , P. Ifju , P. K. Kreski , A. K. Varshneya , J. Eur. Ceram. Soc. 2012, 32, 1551.

[42] G. I. Shim , S. H. Kim , H. W. Eom , D. L. Ahn , J. K. Park , S. Y. Choi , Compos. Part B 2015, 77, 169.

[43] G. X. Gu , M. Takaffoli , M. J. Buehler , Adv. Mater. 2017, 29, 1700060. 10.1002/adma.201700060 28556257

[44] A. Wat , C. Ferraro , X. Deng , A. Sweet , A. P. Tomsia , E. Saiz , R. O. Ritchie , Small 2019, 15, 1900573. 10.1002/smll.201900573 31131997

[45] K. Zhang , Q. Gao , J. C. Jiang , M. S. Chan , X. Y. Zhai , L. C. Jin , J. F. Zhang , J. F. Li , W. H. Liao , Compos. Sci. Technol. 2024, 249, 110475.

[46] F. Chen , Z. H. Tang , Y. Zhu , J. Q. Deng , Y. Q. Li , Y. Q. Fu , S. Y. Fu , Compos. Struct. 2024, 330, 117829.

[47] S. Liu , S. Wang , M. Sang , J. Y. Zhou , J. S. Zhang , S. H. Xuan , X. L. Gong , ACS Nano 2022, 16, 19067. 36302097 10.1021/acsnano.2c08104

