# Supplementary material for: Hybridizing Shear‐Stiffening Gel and Chemically‐Strengthened Ultrathin Glass Sheets for Flexible Impact‐Resistant Armor
Source: Adv Sci (Weinh). 2024 Jun 28;11(33):2403379. doi: 10.1002/advs.202403379 (PMC11434028; doi:10.1002/advs.202403379)
Supplement: Supplementary file 1 — Supporting Information [file ADVS-11-2403379-s002.docx]

**Hybridizing shear-stiffening gel and chemically-strengthened ultrathin glass sheets for flexible impact-resistant armor**

Xuchao Wang, Zijing Zhang, Zhihua Liang, Haimin Yao**^[[1]](#footnote-1)^**

Department of Mechanical Engineering, The Hong Kong Polytechnic University, Hung Hom, Kowloon, Hong Kong SAR, China


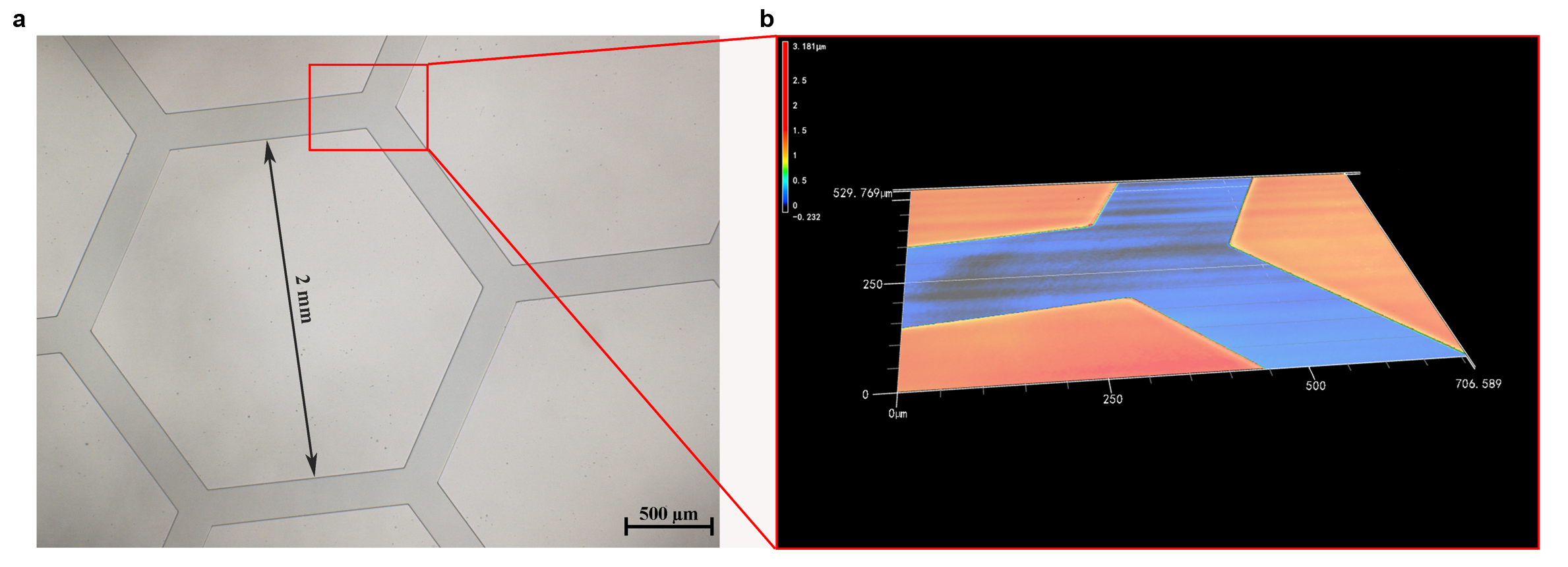


Fig. S1. Hexagonal patterns on the surface of the ultra-thin glass sheet obtained by photoetching process. (a) Optical microscope image showing the hexagonal patterns with size of 2 mm. (b) Surface profile showing the thickness of photoresist.


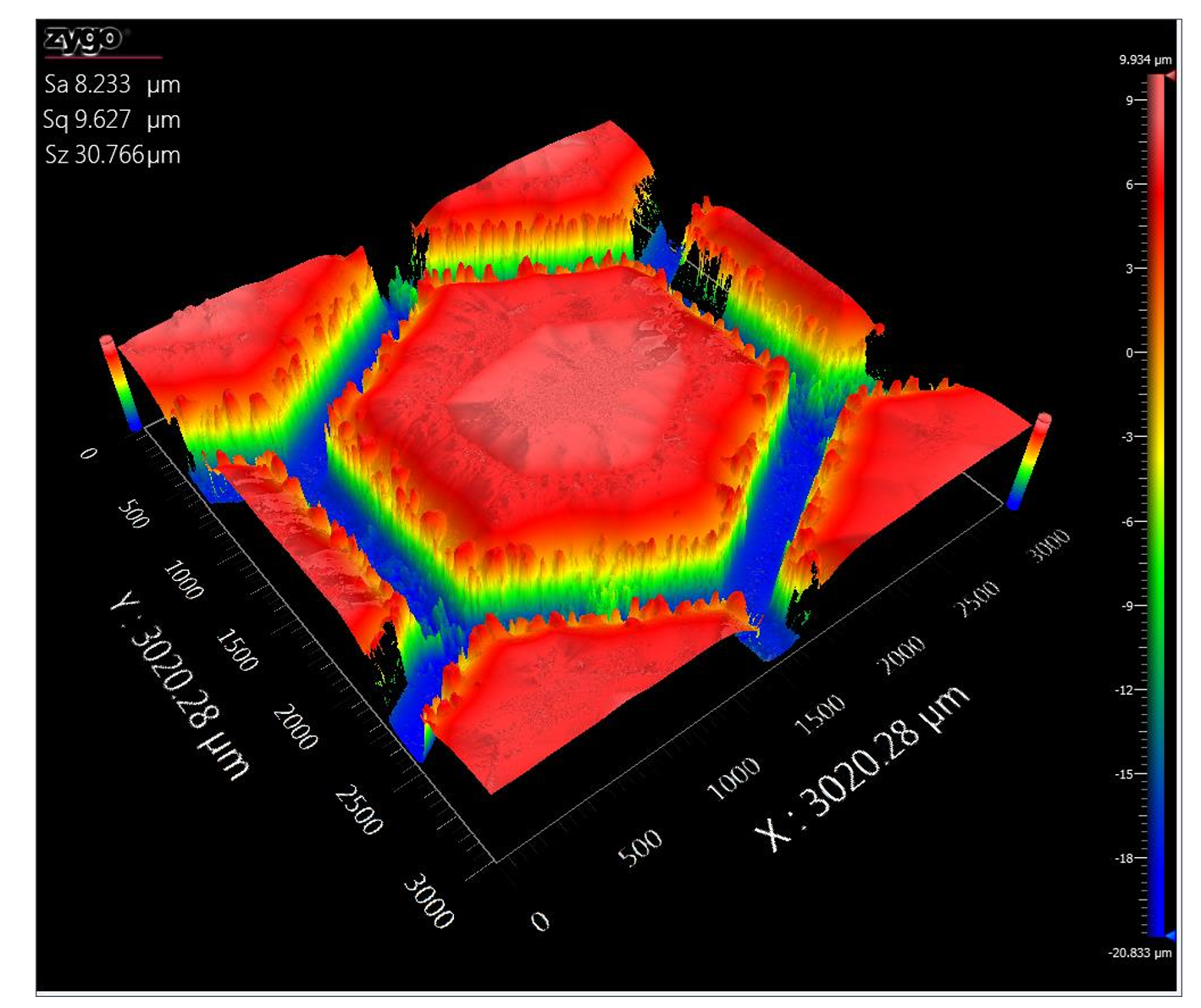


Fig. S2. Surface profile showing morphology and groove depth of the hexagonal islands after chemical etching process.


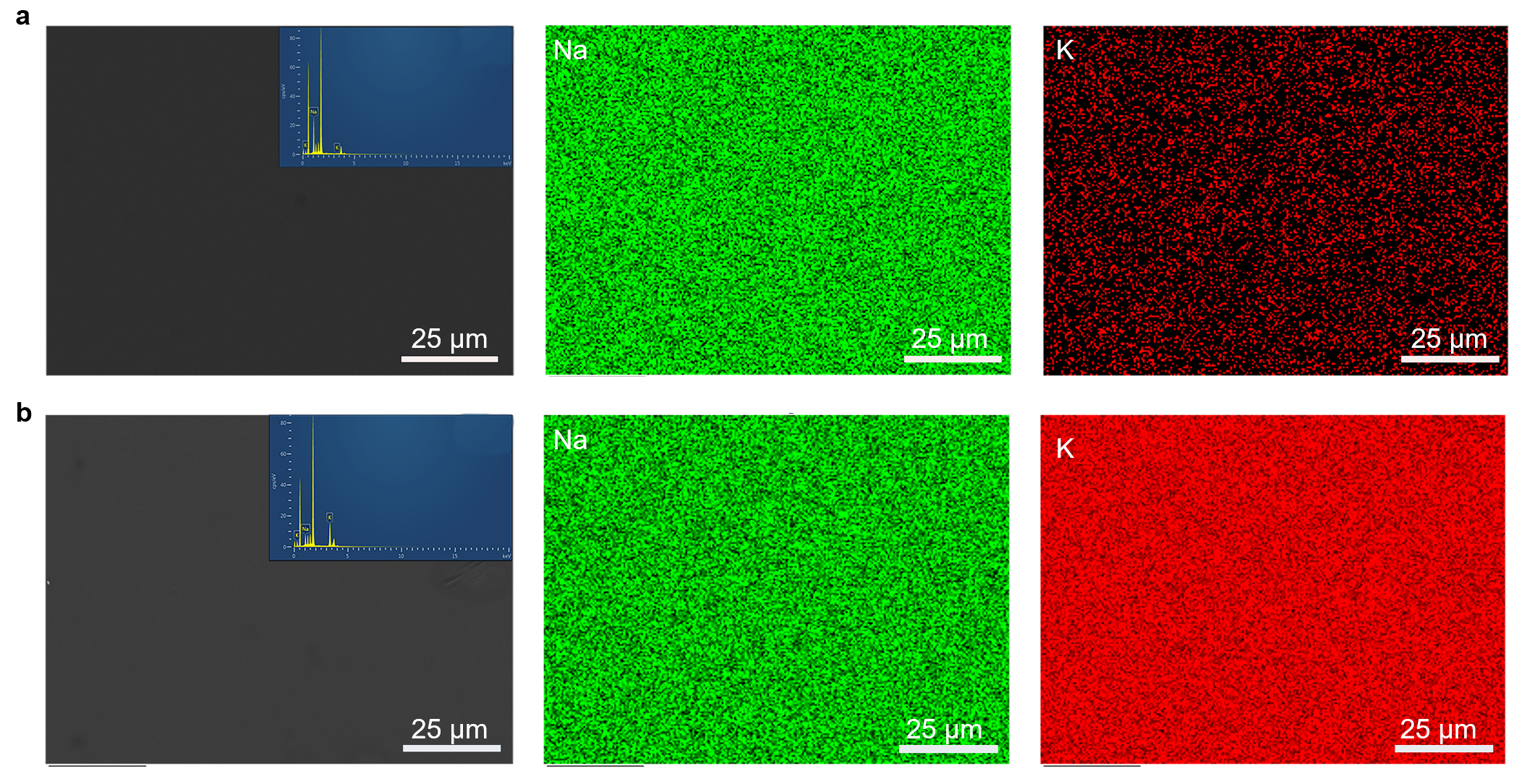


Fig. S3. EDS mapping of (a) the plain glass sheet and (b) the GSGS.


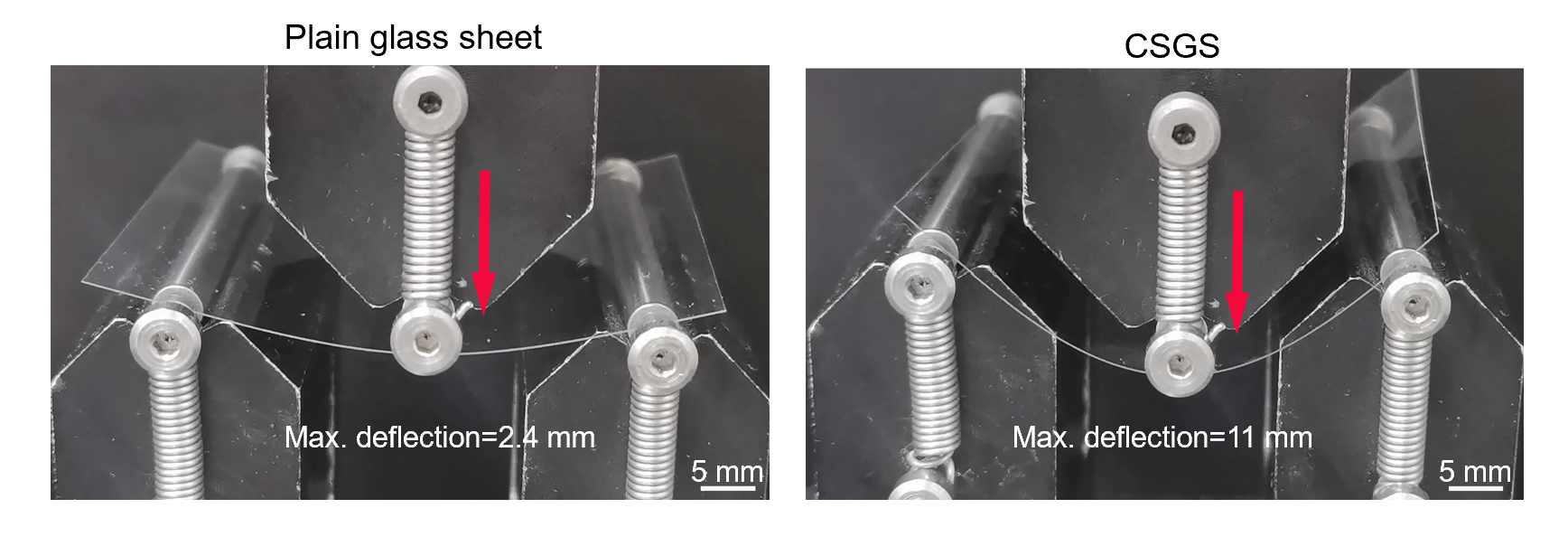


Fig. S4. Flexibility of the plain glass sheet and the GSGS under flexural tests.


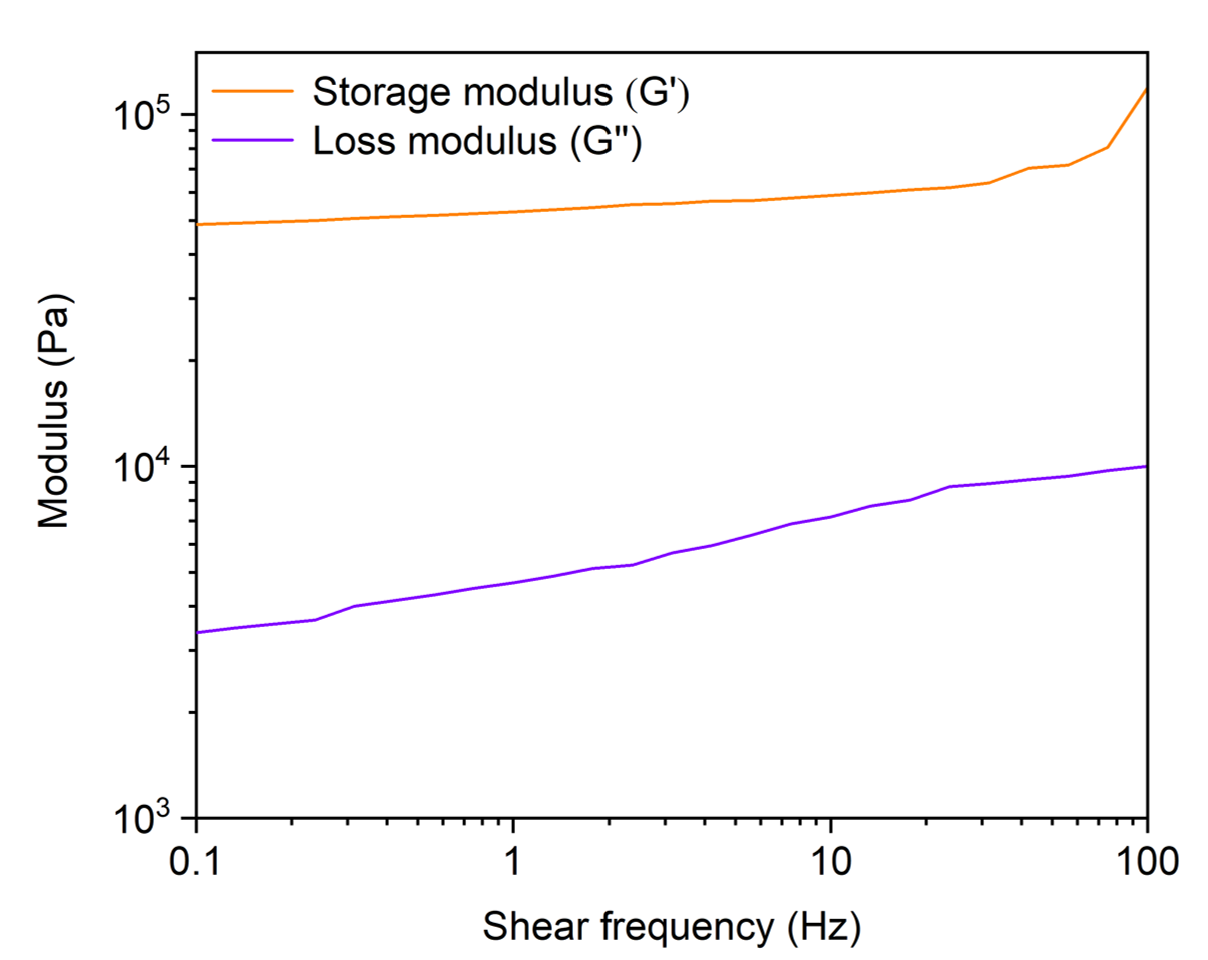


Fig. S5. Rheological properties of PDMS.


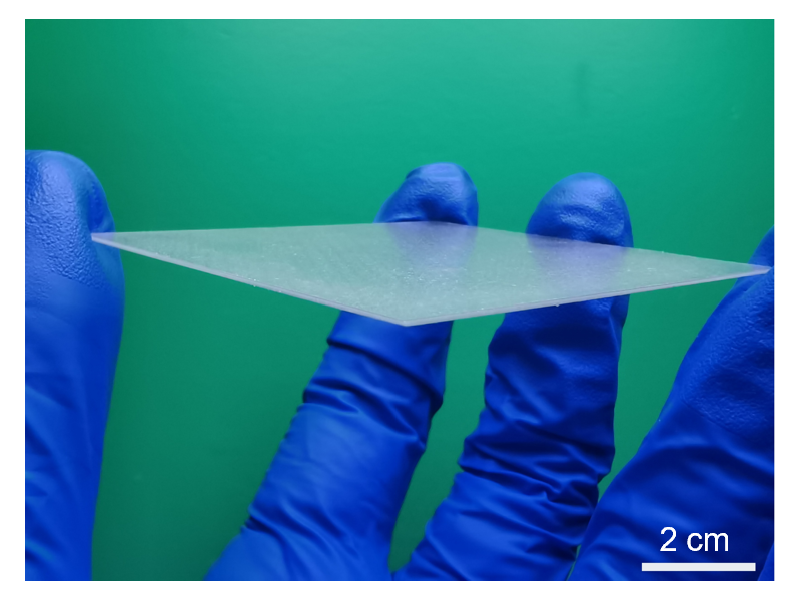


Fig. S6. Digital photograph of PCCL.


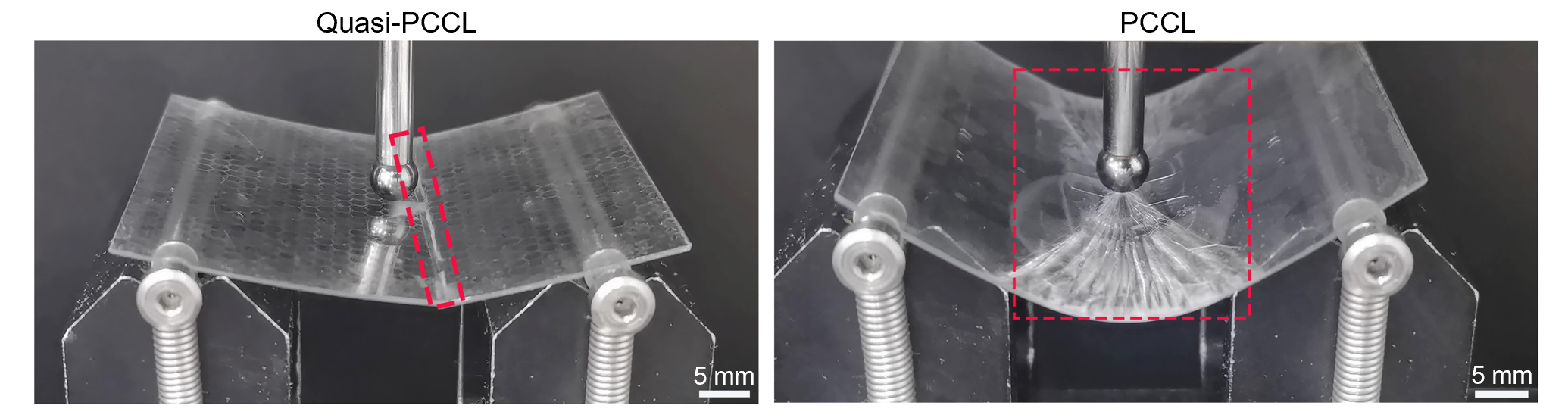


Fig. S7. Failure morphologies in quasi-PCCL and PCCL under quasi-static compression tests.


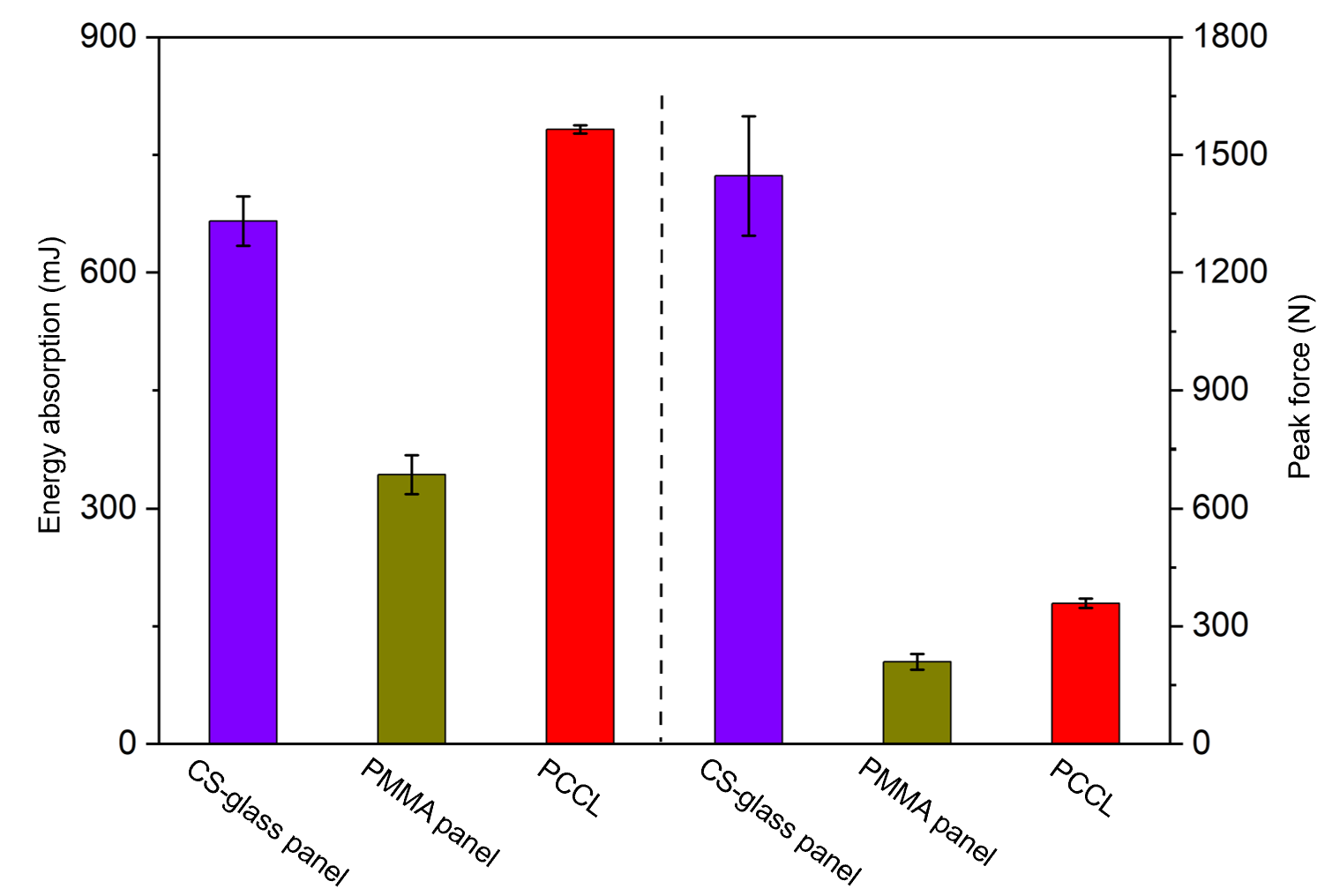


Fig. S8. Comparison of energy absorption and peak force for CS-glass panel, PMMA panel and PCCL under drop-hammer impact tests.


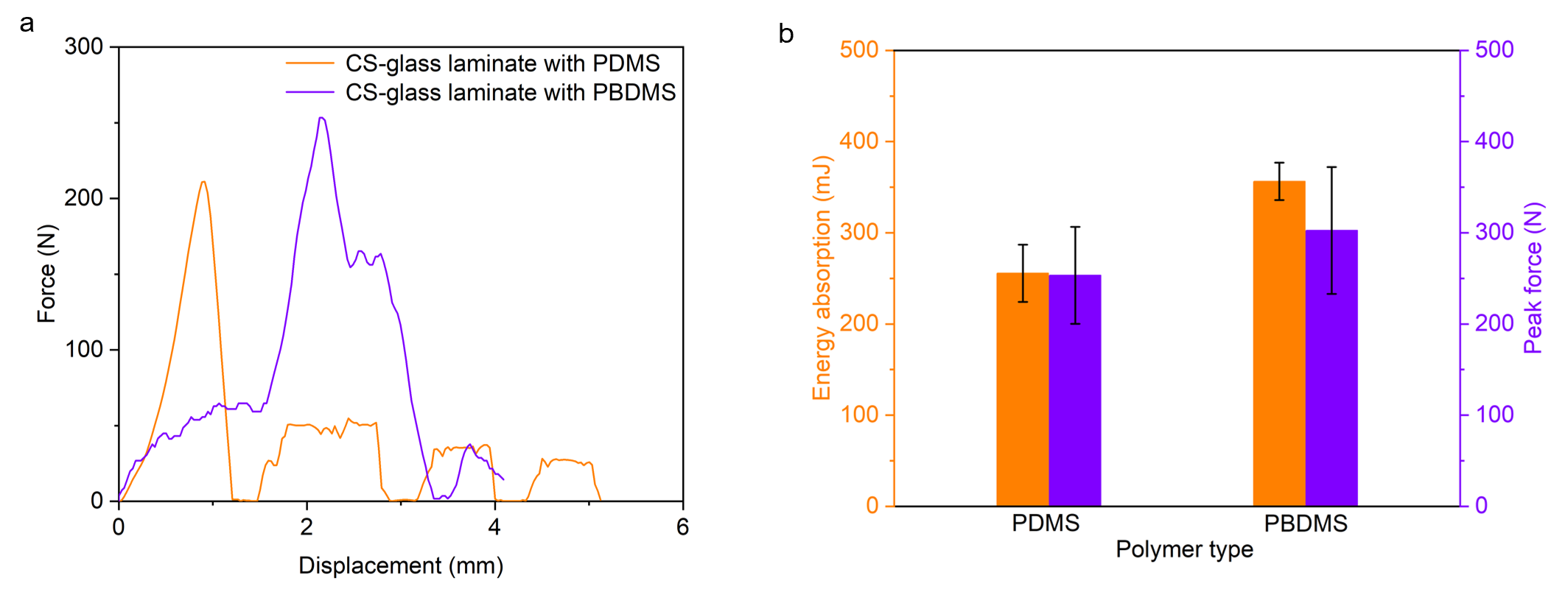


Fig. S9. Investigation of the contribution of PBDMS in improving the impact resistance. (a) Force-displacement curves of the CS-glass laminates with PDMS or PBDMS as the interlayers. (b) Comparison of energy absorption and peak force of the CS-glass laminate with PDMS or PBDMS as the interlayers.

**Table S1.** Compositions of the ultra-thin glass sheets

| Chemical compositions | Contents (wt.%) |
| --- | --- |
| SiO_2_ | 65.85 |
| CaO | 12.65 |
| Na_2_O | 10.65 |
| Al_2_O_3_ | 6.47 |
| MgO | 3.96 |
| K_2_O | 0.13 |
| SO_3_ | 0.12 |
| Fe_2_O_3_ | 0.12 |
| ZrO_2_ | 0.05 |

1. Corresponding author

   E-mail address: mmhyao@polyu.edu.hk (H. Yao) [↑](#footnote-ref-1)
